# Study protocol for Ketamine as an adjunctive therapy for major depression (2): a randomised controlled trial (KARMA-Dep [2])

**DOI:** 10.1186/s12888-023-05365-9

**Published:** 2023-11-16

**Authors:** Ana Jelovac, Cathal McCaffrey, Masashi Terao, Enda Shanahan, Enas Mohamed, Emma Whooley, Kelly McDonagh, Sarah McDonogh, Anna Igoe, Orlaith Loughran, Ellie Shackleton, Ciaran O’Neill, Declan M. McLoughlin

**Affiliations:** 1 Department of Psychiatry, Trinity College Dublin, St. Patrick’s University Hospital, James Street, Dublin, D08 K7YW Ireland; 2 Centre for Public Health, Global Research Institute for Health Sciences, Queen’s University Belfast, Belfast, Northern Ireland United Kingdom; 3 Trinity College Institute of Neuroscience, Trinity College Dublin, Dublin, Ireland

**Keywords:** Depression, Ketamine, Midazolam, Clinical trial, Relapse, Cost effectiveness

## Abstract

**Background:**

Depression is a common psychiatric disorder and a leading cause of disability worldwide. Conventional monoaminergic antidepressants have limited efficacy and take weeks to exert a therapeutic effect. Single infusions of subanaesthetic doses of ketamine exhibit rapid antidepressant action but effects are transient and relapse is common. One potential strategy for increasing ketamine’s antidepressant efficacy and/or prolonging its therapeutic benefit may be serial infusions. There is limited evidence on the efficacy and safety of repeated ketamine infusions against an active comparator.

**Methods:**

This protocol describes an ongoing pragmatic, randomised, controlled, parallel-group, patient- and rater-blind, superiority trial. Eligible adult inpatients with a confirmed DSM-5 diagnosis of a major depressive episode (unipolar or bipolar) are randomly allocated in a 1:1 ratio to a course of up to eight infusions of ketamine or midazolam twice-weekly over four weeks. The primary objective is to assess the efficacy of serial adjunctive ketamine infusions versus active comparator midazolam by measuring Montgomery-Åsberg Depression Rating Scale score difference between arms from before the first infusion to 24 h after the final infusion, supplemented by a 95% confidence interval. To facilitate generalisability of results, the trial takes place under “real world” conditions with both groups continuing to receive regular inpatient care including treatment-as-usual pharmacotherapy, nursing care, and psychological and other therapies during the randomised treatment phase and regular outpatient care thereafter. Participants are monitored for relapse during a 24-week follow-up after the end of the randomised phase. Secondary objectives of the trial are to assess: response and remission rates at the end of randomised phase; relapse status during the 24-week follow-up after the end of the randomised phase; the safety and tolerability of repeated ketamine infusions regarding psychotomimetic and other psychiatric side effects, cognitive side effects, as well as withdrawal symptoms, haemodynamic stability, neurological, urological, and other physical side effects; and quality of life and cost-effectiveness.

**Discussion:**

There is an unmet clinical need for rapidly-acting novel antidepressants. This trial will provide efficacy, safety and health economic data on serial ketamine infusions and thus help inform clinical practice on the potential role of this treatment in the management of depression.

**Trial registration:**

EudraCT 2019-003109-92. Registered 2 October 2019. ClinicalTrials.gov NCT04939649. Registered 25 June 2021.

## Background

Depression is a common psychiatric disorder with a lifetime prevalence of 20% [1] and is a leading cause of disability worldwide [2]. Much of the economic and social burden of depression is attributable to treatment resistance [3]. Despite intensive research efforts, the mainstay of pharmacological treatment for depression over the past 60 years has remained focused mainly on monoamine neurotransmitters. The landmark Sequenced Treatment Alternatives to Relieve Depression (STAR*D) Trial [4] found that only ~ 30% of patients achieved remission after first-line treatment and up to a third did not respond to multiple sequential treatment steps. Aside from their limited effectiveness, conventional monoaminergic antidepressants can take weeks to exert a therapeutic effect, highlighting the need for novel rapidly-acting treatments. One such approach might be the dissociative anaesthetic ketamine.

Ketamine, an antagonist of the N-methyl-D-aspartate receptor (NMDAR) targeting the excitatory neurotransmitter glutamate [5], is a routinely used and relatively inexpensive anaesthetic typically administered intravenously (IV) with a short half-life (2–3 h). Single, slowly administered, subanaesthetic ketamine infusions elicit rapid, though transient, antidepressant response and target core symptoms of depression, including suicidal ideation [6, 7]. A 40-minute 0.5 mg/kg of body weight IV infusion has been the most effective dose to date for both unipolar and bipolar depression, with lower doses having less of an antidepressant effect and higher doses causing more dissociative side effects [8]. Other methods to administer ketamine are being evaluated, including intramuscular and subcutaneous injections, oral ingestion, and intranasal sprays. While these methods are easier to administer than slow infusions, they may be less predictable regarding bioavailability. Together, these findings represent a paradigm shift away from conventional slow-acting monoaminergic antidepressants to a potential new era of rapid-acting antidepressants. However, a definitive role for ketamine in the management of depression is not yet agreed upon and there are legitimate concerns regarding its long-term efficacy and safety [9].

The majority of randomised trials to date have used single ketamine infusions demonstrating robust but transient antidepressant effects, lasting approximately a week. Recent Cochrane reviews of ketamine in unipolar [10] and bipolar depression [11] found it to be more efficacious than placebo 24 h after an infusion. However, the effect size for ketamine is attenuated in trials using an active comparator (midazolam) compared to those using saline placebo [12]. Midazolam itself is not considered an antidepressant medication but is sometimes used in ketamine trials to help maintain blinding because it has acute sedative effects and a similar half-life to ketamine [13]. Midazolam-controlled trials may thus provide a more realistic estimate than saline of the antidepressant effect size of ketamine (Cohen’s *d* = 0.7 in a meta-analysis of ketamine vs. midazolam trials compared to *d* = 1.8 in ketamine trials with saline placebo) [12].

Aside from its potential role in the acute management of suicidal ideation [7], it is unlikely that the transient effects of single ketamine infusions are of practical therapeutic value in the treatment of depression. One potential strategy for increasing ketamine’s antidepressant efficacy and/or prolonging its therapeutic benefit may be repeated infusions administered over the course of several weeks. Several open-label studies have examined serial ketamine infusions but only a handful of randomised trials have been reported to date. Singh et al. [14] randomised patients with depression to twice- or thrice-weekly ketamine infusions or saline and found similar efficacy of the two ketamine groups over saline placebo. More recently, randomised trials have compared serial ketamine infusions to active comparators midazolam [15] and electroconvulsive therapy (ECT) [16, 17]. In military veterans with depression, Shiroma et al. [15] found that serial ketamine was more effective than serial midazolam after five infusions but there was no longer a significant effect of serial ketamine after the sixth infusion during which the midazolam group was crossed over to receive a single infusion of ketamine. In a large unblinded noninferiority randomised trial, Ekstrand et al. [16] showed that remission rates were significantly higher after a course of ECT compared to repeated ketamine infusions, though relapse rates were similar across the one-year follow-up phase. A recent meta-analysis of six randomised trials comprising 340 patients found a significant advantage for ECT over ketamine with a standardised mean difference of 0.69 but this analysis was limited by low to moderate quality of the trials and underpowered research designs [17]. Since then, an open-label randomised trial found that a course of six ketamine infusions was noninferior to ultra-brief pulse ECT in predominantly outpatients with nonpsychotic unipolar depression [18]. However, the interpretation of this finding is complicated by the unusually low remission rate (20%) in the ECT arm.

Given the paucity of data on the efficacy and safety of serial ketamine infusions against an active comparator, there is an unmet clinical need for larger and longer-term parallel group trials. The ongoing KARMA-Dep (2) Trial attempts to address these gaps in knowledge.

### Aims and objectives

The main aim of this trial is to test the primary hypothesis that repeated ketamine infusions as adjunctive therapy to routine care will improve mood outcome in patients hospitalised with depression. We will also test the secondary hypothesis that repeated ketamine infusions will be associated with reduced healthcare costs and improved quality of life.

The primary objective is to conduct a pragmatic randomised controlled patient- and rater-blinded trial of repeated adjunctive twice-weekly ketamine vs. midazolam infusions over a period of up to four weeks (i.e., up to a maximum of eight infusions) for patients hospitalised for depression and to assess the depression score difference between arms from before the first infusion to 24 h after the final infusion.

Secondary objectives are to assess response and remission rates at the end of the randomised treatment phase and relapse status after 24 weeks, assess the safety and tolerability of ketamine vs. midazolam regarding psychiatric, cognitive and physical side effects; and conduct quality of life, cost-effectiveness and cost-utility analyses.

## Methods

### Study design and setting

This Phase III pragmatic, randomised, controlled, patient- and rater-blinded, parallel-group, superiority trial is underway at St Patrick’s University Hospital, a 241-bed inpatient psychiatric facility and university teaching hospital located in central Dublin and its sister facility, the 52-bed St Patrick’s Hospital Lucan located in suburban/semirural County Dublin, Ireland. Both hospitals form part of the national St Patrick’s Mental Health Services (https://www.stpatricks.ie/), Ireland’s largest single independent sector provider of mental health care. The coordinating centre for the trial is St Patrick’s University Hospital. Recruitment, assessments and treatments could only take place at the St Patrick’s University Hospital centre while the second centre (St Patrick’s Hospital Lucan) was closed for new admissions from March 2020 to August 2023 during which time it served as a COVID-19 quarantine facility [19]. The first participant was randomised in September 2021 following delays due to the COVID-19 pandemic. This trial is sponsored by Trinity College Dublin, Ireland (clinicaltrialsponsorship@tcd.ie), who oversee regular monitoring of the trial processes and provide Pharmacovigilance services.

The design of this trial was informed by a pilot trial to assess trial procedures [20]. The pilot trial showed that patient recruitment and follow-up was satisfactory. Following pilot trial experience, there were three main design changes: (1) infusion frequency was increased from once weekly to twice weekly; (2) a second centre (closed from March 2020 to August 2023) was added to enhance recruitment rate; (3) to be in line with the majority of other ketamine trials in depression, we elected to use the Montgomery-Åsberg Depression Rating Scale (MADRS) instead of the Hamilton Rating Scale for Depression.

Eligible consenting participants are randomly allocated in a 1:1 ratio to a course of up to eight infusions of adjunctive ketamine or midazolam twice weekly over four weeks. To facilitate generalisability of results, the trial takes place under “real world” conditions with both groups continuing usual inpatient care (i.e., treatment-as-usual concomitant pharmacotherapy, nursing care, and psychological and other therapies) during the randomised treatment phase and routine outpatient care and review thereafter. Participants are followed up for 24 weeks after the end of the randomised treatment phase to identify if and when relapse occurs. During the allocated infusions and follow-up period, patients are monitored for treatment-related adverse events relating to both mental and physical health.

### Eligibility criteria

This trial is enrolling inpatients with moderate-to-severe unipolar or bipolar depression who meet eligibility criteria (Table 1) at screening Visit 0 and before the first infusion at Visit 1. Electronic healthcare records of all hospital admissions are pre-screened for eligibility by a medical doctor. Potentially eligible participants admitted for treatment of a major depressive episode are approached by a member of the research team and provided with verbal and written information (Participant Information Leaflet) about the study. If agreeable to participate, written informed consent is obtained by a medical doctor and more detailed screening is carried out to confirm eligibility.

**Table 1:** Inclusion and exclusion criteria

Inclusion criteria	1. Adult (aged ≥ 18 years) male or female voluntary admission able and willing to give written informed consent and comply with the requirements of this study protocol
	2. Admitted to hospital and diagnosed with major depressive disorder or bipolar disorder (current episode depression), confirmed by the MINI and have a MADRS score ≥ 20 at screening and start of the first infusion
	3. Female patients of child-bearing potential and male patients whose partner is of child-bearing potential must be willing to ensure that they or their partner use two contraception methods, including a barrier method, during the randomised treatment phase and for 12 weeks thereafter
	4. Female patients’ plasma pregnancy test performed at the screening visit must be negative
	5. Patients have clinically acceptable laboratory and ECG findings during the current admission prior to the first infusion session
Exclusion criteria	1. Current involuntary admission
	2. Patients unable to provide written informed consent
	3. Patients who have participated in another ketamine study or received any other investigational agent within the past 12 months
	4. Medical condition rendering unfit for ketamine or midazolam (Ketamine is contraindicated, as per Summary of Product Characteristics, in persons in whom an elevation of blood pressure would constitute a serious hazard. Ketamine should not be used in patients with eclampsia or pre-eclampsia, severe coronary or myocardial disease, CVA or cerebral trauma or if there is hypersensitivity to the active substance. Contraindications to midazolam include known hypersensitivity to benzodiazepines, severe respiratory failure or acute respiratory depression. Bradycardia is a known adverse effect of midazolam and ketamine. Therefore, patients with pre-existing bradycardia are excluded.)
	5. Currently taking any of the following contraindicated medications: ketoconazole, voriconazole, itraconazole, fluconazole, erythromycin, telithromycin, clarithromycin, saquinavir, nefazodone, diltiazem, verapamil, theophylline
	6. Active suicidal intention (score of 6 on item 10 [Suicidal Thoughts] on the MADRS)
	7. Confirmed diagnosis of dementia
	8. Lifetime history of schizophrenia or schizoaffective disorder; active anorexia nervosa or bulimia nervosa in the past 12 months; alcohol or other substance use disorder (with the exception of nicotine) in the previous six months; any DSM-5 disorder other than a major depressive episode (unipolar or bipolar) as the primary presenting problem
	9. ECT administered within the last two months
	10. Pregnancy or inability to confirm use of adequate contraception during the trial
	11. Breastfeeding women

*CVA* Cerebrovascular accident, *DSM-5* Diagnostic and Statistical Manual of Mental Disorders, Fifth Edition, *ECG* Electrocardiogram, *ECT* Electroconvulsive therapy, *MADRS* Montgomery-Åsberg Depression Rating Scale, *MINI* Mini-International Neuropsychiatric Interview

### Interventions

The investigational medicinal product (IMP) is ketamine (Ketalar 10 mg/ml Solution for Injection/Infusion, Pfizer Ireland; 0.5 mg/kg of body weight) and the active comparator is midazolam (Hypnovel 10 mg/5ml solution for injection, Roche Pharmaceuticals Ireland; 0.045 mg/kg of body weight). Both are made up as colourless saline solutions and administered over 40 min using an infusion pump to deliver the required total amount of ketamine or midazolam as per individual body weight, in a course of up to eight infusions given twice-weekly over four weeks. Midazolam was chosen as the active comparator to help maintain blinding because it has acute sedative effects and a similar half-life to ketamine.

Both groups continue to receive treatment-as-usual concomitant psychotropic and other medications with the exception of contraindicated medications (ketoconazole, voriconazole, itraconazole, fluconazole, erythromycin, telithromycin, clarithromycin, saquinavir, nefazodone, diltiazem, verapamil and theophylline). Medication changes are monitored and recorded throughout the infusion period and at follow-up timepoints. As per Summary of Product Characteristics for Ketalar (ketamine), diazepam is known to increase the half-life of ketamine and prolongs its pharmacodynamic effects. Concurrent use of diazepam or other benzodiazepines increases plasma levels and reduces the clearance rate of ketamine. However, benzodiazepines do not appear to interact with the antidepressant effect of ketamine [15]. Where possible, patients taking any regular benzodiazepines should omit their dose on the morning of infusion sessions. It is appreciated that omission of benzodiazepines may not be possible for all patients. This will be as per the Investigator’s discretion. Patients may also avail of psychotherapies, occupational therapy, psychoeducation programmes and other non-pharmacological interventions as part of their usual inpatient care. This reflects the conditions under which adjunctive ketamine would be used in routine clinical inpatient practice, thereby enhancing the generalisability and external validity of the trial.

Infusions are discontinued early if there are persisting nonphysiological haemodynamic changes (i.e. heart rate > 110/minute or systolic/diastolic blood pressure > 180/100 for more than 15 min) that do not respond to beta-blocker therapy. Such events are discussed with the Principal Investigator and reported to the Sponsor. Patients are withdrawn from the randomised phase of the trial if: (i) an infusion is discontinued for the above haemodynamic reasons or other serious medical contraindications (e.g., oversedation, hypoxia, intolerable adverse physical reactions); (ii) the patient develops mania or psychosis; (iii) the patient becomes severely depressed and/or suicidal. The Principal Investigator assesses the details of the withdrawal and the Sponsor is notified. In the event of early discontinuation of treatment within the randomised phase of the trial, the Investigators inform patients and ensure that the 24-week follow-up after the last infusion is arranged.

In this study, interventions are administered intravenously by the trial anaesthetist and thus there is limited opportunity for non-compliance with regards to actual treatment with the IMPs. The Investigators are responsible for ensuring that the study treatment is administered in compliance with the protocol. Patient compliance is assessed by maintaining dispensing records.

Should the patient require medical investigation and/or treatment due to an overdose of study treatment, cost will be covered by the trial indemnity policy, unless due to negligence or malpractice. Insurance for this trial is provided by indemnity cover for research in place at both Trinity College Dublin (Sponsor) and St Patrick’s Mental Health Services. Physicians involved in the trial have medical malpractice insurance.

### Efficacy outcomes

The primary outcome is the change in the clinician-rated Montgomery-Åsberg Depression Rating Scale (MADRS) [21] score from baseline to 24 h after the final infusion.

Secondary efficacy outcomes are:

i. change in subjective mood from baseline to 24 h after the final infusion on the patient-rated Quick Inventory of Depressive Symptoms, Self-Report (QIDS-SR_16_) [22].
ii. response (defined as ≥ 50% improvement in MADRS score from baseline to 24 h after the final infusion [23])
iii. remission (defined as MADRS score ≤ 10 at 24 h after the final infusion [23, 24])
iv. relapse (in treatment remitters, relapse is defined as a MADRS score of ≥18 at any given follow-up timepoint. MADRS mood ratings are repeated at weeks 6, 12 and 24 during the follow-up period after the final infusion. Hospital readmission and deliberate self-harm/suicide also constitute relapse and timing of such events is recorded. Where relapse occurs following hospital discharge and whilst an outpatient, the patient’s clinical team is informed.)

### Secondary safety and tolerability outcomes

Secondary safety and tolerability outcomes consist of psychotomimetic, dissociative, cognitive and physical health effects of repeated ketamine infusions, measured before, during and after infusions using a range of validated instruments described below.

### Economic and quality of life outcomes

Resource use is collected and other healthcare costs estimated using a version of the Client Service Receipt Inventory (CSRI) adapted for a recent antidepressant trial and cost-effectiveness study [25]. Health-related quality of life is measured using the 5-level version of the EuroQol five-dimensional questionnaire (EQ-5D-5L) [26, 27].

### Assessments

Table 2 provides a schematic summary of timings of study assessments and procedures. Where additional study visits are clinically indicated, an unscheduled visit is performed. At screening visit, demographic and clinical characteristics of participants are recorded, as well as their medical and surgical history. A physical examination is performed and includes the evaluation of the cardiovascular, dermatological, musculoskeletal, respiratory, gastrointestinal and neurological systems. Height, weight and temperature are also recorded. Several psychiatric assessments are also carried out. Copies of all assessments and relevant trial documents are located in the Investigator Site File.

**Table 2:** Schedule of trial assessments and procedures

		Inpatient								Follow-up		
		Treatment										
Assessment	Screening Visit 0	Visit 1	Visit 2	Visit 3	Visit 4	Visit 5	Visit 6	Visit 7	Visit 8/Final Infusion Visit	Visit 9	Visit 10	Visit 11
		Week 1		Week 2		Week 3		Week 4		6 weeks± 1 week	12 weeks± 2 weeks	24 weeks ± 3 weeks
Informed Consent	X											
Inclusion/Exclusion criteria	X	X	X	X	X	X	X	X	X			
Past and current medical conditions	X											
Demographics	X											
MINI, ATRQ	X											
Physical exam	X											
Height, weight, BMI	X	X		X		X		X				
ECG		X	X	X	X	X	X	X	X			
Clinical laboratory assessments	X								X			
Blood pregnancy test	X											
Vital signs		X	X	X	X	X	X	X	X			
Randomisation	X											
Treatment review		X	X	X	X	X	X	X	X	X	X	X
Drug administration		X	X	X	X	X	X	X	X			
MADRS	X	X	X	X	X	X	X	X	X	X	X	X
QIDS-SR_16_	X	X	X	X	X	X	X	X	X	X	X	X
MoCA	X	X							X		X	X
OAA/S-R		X	X	X	X	X	X	X	X			
CADSS		X	X	X	X	X	X	X	X			
BPRS		X	X	X	X	X	X	X	X			
YMRS		X	X	X	X	X	X	X	X			
PRISE	X	X	X	X	X	X	X	X	X	X	X	X
AE review		X	X	X	X	X	X	X	X	X	X	X
SAE review		X	X	X	X	X	X	X	X	X	X	X
PWC-20	X				X				X	X	X	
Concomitant medication	X	X	X	X	X	X	X	X	X			
Healthcare costs: CSRI, medications costs	X									X	X	X
EQ-5D-5L	X									X	X	X
Drug accountability		X	X	X	X	X	X	X	X			
Assessment of patient and rater blinding		X							X			X
Fasting		X	X	X	X	X	X	X	X			

*MINI* Mini-International Neuropsychiatric Interview, *ATRQ* Antidepressant Treatment Response Questionnaire, *BMI* Body mass index, *ECG* Electrocardiogram, *MADRS* Montgomery-Åsberg Rating Scale for Depression, *QIDS-SR*_16_ Quick Inventory of Depressive Symptoms, *MoCA* Montreal Cognitive Assessment, *OAA/S-R* Observer’s Assessment of Alertness/Sedation Scale - Responsiveness Subscale, *CADSS* Clinician-administered Dissociative States Scale, *BPRS* Brief Psychiatric Rating Scale, *YMRS* Young Mania Rating Scale, *PRISE* Patient-Rated Inventory of Side Effects, *AE* Adverse event, *SAE* Serious adverse event, *PWC-20* 20-item Physician Withdrawal Checklist, *CSRI* Client Service Receipt Inventory, *EQ-5D-5L* EuroQol-5 dimensions-5 level scale for health status

Participants are followed up over 24 weeks to assess for relapse. Follow-up mood, safety outcome and health economics assessments, as well as recording of concomitant medications, are repeated at weeks 6, 12 and 24 as outlined in Table 2. Where in-person follow-up appointments are not possible, assessments can take place over the telephone or by videoconference where practicable. Reasonable meal and travel expenses incurred by participants attending follow-up appointments are reimbursed.

#### Psychiatric assessments

The Mini-International Neuropsychiatric Interview (MINI) [28] is a validated and reliable [29] structured diagnostic interview for Diagnostic and Statistical Manual of Mental Disorders, Fifth Edition (DSM-5) disorders administered at screening visit to confirm a clinical diagnosis of a major depressive episode occurring within the context of major depressive disorder or bipolar disorder. The presence of treatment-resistant depression is ascertained using the Antidepressant Treatment Response Questionnaire (ATRQ) [30].

The Montgomery-Åsberg Depression Rating Scale (MADRS) [21] is a clinician-rated depression rating scale performed at screening visit and repeatedly throughout the trial as a measure of depressive symptomatology and response to treatment. To enter the study, patients must score ≥ 20 at Visit 0 (Screening) and prior to the first infusion at Visit 1. Sleep and appetite scores from items 4 and 5 are carried over from − 40 min before each infusion to + 60 min, + 120 min, and 24 h after each infusion in line with the methodology of previous studies [31–34]. MADRS interviews are performed by trained assessors using a structured interview guide with reported interrater reliability of 0.93 [35]. Outcome assessors (psychiatrists, registered nurses and research assistants) are trained in the administration of the MADRS by the Investigators. Training is repeated every six months using videotaped MADRS interviews with formal assessment of interrater reliability.

The Quick Inventory of Depressive Symptoms, Self-Report (QIDS-SR_16_) [22] is a validated self-report measure of depressive symptoms. Sleep and appetite items (1, 2, 3, 4, 6, 7, 8 and 9) on the QIDS-SR_16_ are carried over from − 40 min before each infusion to + 60 min, + 120 min and 24 h after each infusion in line with the methodology of previous studies [31–34]. QIDS-SR_16_ has high internal consistency (Cronbach’s α = 0.86) and concurrent validity with the 24-item Hamilton Rating Scale for Depression (*r* = 0.86) [22].

#### Cognitive assessments

The Montreal Cognitive Assessment (MoCA) [36] is performed at screening visit and several subsequent visits to assess cognitive function. The MoCA is a rapid screening instrument for mild cognitive dysfunction assessing the following cognitive domains: attention and concentration, executive functions, memory, language, visuoconstructional skills, conceptual thinking, calculations, and orientation. Parallel versions are administered in a counterbalanced order at different visits to minimise practice effects. The MoCA has good test-retest reliability (*r* = 0.92), internal reliability (α = 0.83) and content validity (*r* = 0.87 correlation with Mini-Mental State Examination) [36].

#### Physical assessments

Participants’ vital signs (heart rate, pulse oximetry and blood pressure) are monitored during each infusion clinic before, during and up to + 120 min after the start of an infusion. A resting 12-lead electrocardiogram (ECG) is obtained routinely during the current admission prior to the first infusion session. Continuous ECG monitoring is performed throughout the infusion observation period. Recent routine laboratory investigations (i.e. full blood count, renal, liver and thyroid function tests) obtained during the current admission are reviewed by an Investigator who is a medical doctor and trial anaesthetist prior to the first infusion to ensure the patient is medically stable. Liver function tests are repeated after the final infusion; if abnormal, they are repeated until normalisation or throughout the 24-week follow-up period. Blood serum pregnancy tests are performed at screening visit before first infusion for women of childbearing potential. The results must be available, documented and negative before the first dose of study drug is administered. Date of last menstrual period is documented. Information relating to the importance of contraception during the trial is provided in the Participant Information Leaflet.

#### Prior and concomitant medication

All over-the-counter or prescription medication, vitamins, herbal supplements and any other therapies in the previous one month are recorded at screening visit. Changes in medications and therapies are monitored and documented at all assessment time points.

#### Safety and tolerability assessments

During each infusion, adverse or psychotomimetic effects of either ketamine or midazolam are monitored using validated scales comprising: Observer’s Assessment of Alertness/Sedation Scale - Responsiveness Subscale (OAA/S-R) [37]; Clinician-Administered Dissociative States Scale (CADSS) [38]; Brief Psychiatric Rating Scale (BPRS) [39]; Young Mania Rating Scale (YMRS) [40]; and the Patient-Rated Inventory of Side Effects (PRISE) [41]. These are administered before, during and/or after infusions in order to capture the range of possible subjective and objective side effects of either agent.

One concern about ketamine use for treating depression is the potential for development of substance use disorder [9]. The 20-item Physician Withdrawal Checklist (PWC-20) [42] is used to assess for potential withdrawal symptoms during and after completing the allocated course of ketamine or midazolam infusions. The PWC-20 is a brief, easy-to-use instrument and has been reported to have good validity, internal consistency, test-retest and interrater reliability [42].

The adverse events reporting period is from the time of consent to the end of the 24-week follow-up period. At each visit, participants are asked if they have experienced any adverse or serious adverse events since the last visit. All adverse medical, psychotomimetic and general events are reported to the Data Monitoring and Trial Steering Committees. Serious adverse events are reported to the Pharmacovigilance service provided by the Sponsor at the Wellcome-Health Research Board Clinical Research Facility at St James’s Hospital, Dublin, Ireland.

### Data management

Central data management is performed by the Data Management Centre at the Health Research Board Clinical Research Facility at University of Galway, Ireland (https://www.universityofgalway.ie/hrbcrfg/services/informationsystemsdata/). Local user access to the electronic case report forms is controlled via assigned usernames and passwords, approved by the study Data Manager based at University of Galway. Access to the central study database is governed by the Health Research Board Clinical Research Facility at University of Galway Standard Operating Procedures and signed off by the Lead Site Investigator. Audit trails log all transactions of data into and out of the system including time, date, user ID and the records involved. All external electronic communication with the central database are protected by using Secure Socket Layer technology. The main database is hosted in a secure enterprise scale data centre.

Once registered to the trial, the patient is provided with a unique, study-specific participant identifier number and this is the only way the patient is identified in the database. Data are directly entered into the Clinical Data Management System by the site staff. To promote data quality, data entry is by double data entry. Range checks for data values are incorporated into the data entry system.

Personal data are securely stored at the coordinating centre at St Patrick’s University Hospital. To protect confidentiality, personal data do not leave the coordinating centre and only pseudonymised data are entered into the Clinical Data Management System. Data will be retained for 25 years in accordance with Clinical Trial Regulation (CTR) (EU Regulation 536/2014). Access to the final trial dataset will be limited to the named Investigators.

### Sample size calculation

The original power calculation was based on a previous randomised trial of adjunctive serial ketamine infusions versus saline by Singh et al. (2016) [14]. Forty-one patients are required per initial randomisation group (*n* = 82) to have 90% power to demonstrate, using a two-sided t-test at 5% level, that mean change in MADRS score in the ketamine group will be ≥ 8 points that achieved in the midazolam group. This calculation conservatively assumes a standard deviation for the change in mean MADRS of 11, and that the assumptions of a t-test are broadly met, which is expected to be the case with approximately 40 patients per group. Anticipating a 20% withdrawal rate, the recruitment target is 52 patients per group (total *n* = 104). A between-group difference of 6–8 points on the MADRS is an estimate of the minimal important difference derived from the clinical anchor of the difference between full and partial remission in depression [43].

### Randomisation, blinding and allocation concealment

Participants who meet eligibility criteria and who provide written informed consent are randomly assigned to one of two treatment groups in a 1:1 ratio. Study treatment assignment is blinded for participants, their healthcare providers, outcome assessors and data analysts. To ensure patient safety during infusions and in the post-infusion period, the trial anaesthetist administering the infusions is not blinded but is not involved in outcome assessments or data analysis. Random allocation, using randomly permuted blocks was done independently by the Centre for Support and Training in Analysis and Research, University College Dublin, Ireland (http://www.cstar.ie/).

To ensure allocation concealment, allocation information is provided in a randomisation list available only to the trial anaesthetist and trial pharmacist and delivered by secure mail. This information is contained in sequentially numbered opaque sealed envelopes which are stored in a locked clinic office within a locked box to which only the trial anaesthetist has the key. A matching set of opaque randomisation envelopes is also stored in a locked drawer in the locked office of the Assistant Director of Nursing at St Patrick’s University Hospital, to be accessed by clinical staff in the event of emergency unblinding. The Pharmacovigilance team also has a matching set of opaque randomisation envelopes for emergency unblinding. The matching set of envelopes containing allocation information will remain unopened but may be used where emergency unblinding is indicated where, in the opinion of the Investigator or other physician, it is necessary in order to assess and/or treat an adverse event. Unblinding for one or all participants will take place if it is in the best interests of the participants in order to assess and treat an adverse event. In the case of an emergency, when knowledge of the treatment assignment is essential for the clinical management of the patient, any Investigator may unblind a single patient.

### Statistical methods

A complete Statistical Analysis Plan approved by the Data Monitoring and Trial Steering Committees can be found at https://osf.io/t7ukx. There are no plans to conduct an interim analysis.

#### Analysis of primary endpoint

The primary analysis will be conducted once at end-of-trial by a statistician blinded to group labels. To produce this, a general linear model will be fitted to the MADRS scores, with trial arm, site and baseline MADRS score at -40 min prior to the first infusion as covariates. The dependent variable will be the MADRS scores 24 h after the final infusion. The efficacy of the treatment will be evaluated by way of a statistical test of the coefficient for trial arm, the *p*-value for which will be compared to 0.05. The coefficient itself will be presented as the effect size, corresponding to an adjusted MADRS score mean difference between arms after the final infusion, and supplemented by a 95% confidence interval. For a single primary outcome, no adjustment to the type I error is needed.

#### Handling of missing data

The primary analysis will adopt an analytic model using pairwise deletion of the dependent variable (complete case analysis). It is not expected that covariates will have any missing values, as these will comprise randomised arm, site and baseline MADRS score. In the event that more than 5% of participants are missing the MADRS score 24 h after last infusion, multiple imputation by chained equations will be performed using baseline and available subsequent MADRS scores to attempt to recover as much information as possible. The general linear model analysis will be conducted on the imputed data as described above.

#### Analysis of secondary outcomes: response, remission and relapse

No formal subgroup analyses are planned, as low power to detect interactions with subgrouping factors is expected. Binary secondary outcomes of response, remission and relapse will be summarised per treatment arm and descriptively presented.

Similar generalised linear models will be used to describe continuous secondary outcomes (QIDS-SR_16_ and MoCA). As these outcomes were not powered by design, we will avoid claims of statistical significance and focus on interpretation of effect sizes and confidence intervals. *P*-values will be presented to complement this and an adjustment for the False Discovery Rate using the Benjamini-Hochberg approach will be carried out [44]. Future secondary analyses from data collected for this trial will adopt the same approach to multiplicity correction.

Non-continuous secondary outcomes relating to safety and tolerability will be presented descriptively; no formal analyses are planned due to anticipated low power.

Statistical analyses will be performed in R (R Core Team, Vienna, Austria) and Stata 18 (StataCorp, College Station, TX).

#### Health economics and quality of life analyses

The study will adopt a public payer perspective for the main health economics analysis in line with guidance from the Health Information and Quality Authority of Ireland (https://www.hiqa.ie/sites/default/files/2020-09/HTA-Economic-Guidelines-2020.pdf). Healthcare use will be itemised by category, including primary, secondary and community care service use; secondary care will include details on admission as well as the duration of admissions. Details of prescribed medicines will also be collected. Data will cover the entire duration of the study. Care will be monetised using standard references for Ireland as well as recourse to the finance department of St. Patrick’s Mental Health Services and include the acquisition and administration cost of ketamine for the intervention group. Administration costs will be based on staff time and salaries of those involved in its delivery.

Incremental costs, reflecting the cumulative difference in healthcare costs accrued over the study, will be related to incremental effects in a series of cost-effectiveness analyses. In the main economic analysis, between-group differences in MADRS score between the first and final visits (i.e., 24-week follow-up) will be estimated. To allow for the possible joint distribution of costs and effects, incremental cost effectiveness ratios will be based on a bootstrapping exercise undertaken in Microsoft Excel for 1000 bootstrapped estimates of the trial sample. The analysis will be repeated using a seemingly unrelated regression analysis with costs and outcomes as dependent variables and baseline scores and group membership (intervention versus control) as controls. In secondary economic analyses, differences in EQ-5D-5L scores between the first and final visits, as well as area under the curve analyses for MADRS and EQ-5D-5L, will be used to assess outcomes. In the main economic analysis, no discounting will take place given the duration of the study. Data from EQ-5D-5L will be extrapolated to provide an estimate of potential quality-adjusted life year gains based on assumed life expectancy. No subgroup analysis is planned.

Results will be presented as a series of descriptive statistics for control and intervention groups (means and standard deviations). Between-group differences will be presented as differences in means (independent t-tests) and as incremental cost-effectiveness ratios with bootstrapped and regression confidence intervals for the analysis used, as appropriate.

Sensitivity analyses will include a consideration of a societal perspective for costs in which lost productivity related to absences from work, monetised using gross domestic product per capita, will be undertaken and exclusion of high-cost outliers considered. Missing data will be imputed where necessary using multiple imputation methods.

Economic statistical analyses will be performed in Stata 18 and Microsoft Excel.

### Data monitoring and Trial Steering committees

The independent Data Monitoring Committee (DMC) reviews blinded data on a six-monthly basis during the trial and acts according to the DMC Charter, which has been ratified at the organisational meeting. The Charter is stored in the Investigator Site File at the coordinating centre. The composition of the DMC includes an independent statistician and two independent academic psychiatrists. No member of the DMC has a conflict of interest with the Sponsor. Blinded data are presented to the DMC for safety evaluation every six months. Should the DMC wish to review unblinded data, this will be provided by an unblinded statistician who will otherwise not be analysing trial data. The DMC reports to the Trial Steering Committee (TSC), which has authority to decide whether the trial should be suspended or ended.

The Trial Steering Committee (TSC) comprises Investigators, clinical experts not directly involved in the trial, a service user representative, and staff nominated by the Sponsor. The TSC also includes members who are independent of the Investigators, St Patrick’s University Hospital, the funders (Health Research Board) and the Sponsor. The TSC will consider and act, as appropriate, upon the recommendations of the DMC and ultimately carries the responsibility for deciding on premature termination of the trial. The TSC takes responsibility for the scientific validity of the study protocol, assessment of study quality and conduct as well as for the scientific quality of the final study report.

### Auditing

The Principal Investigator will ensure that access will be granted to authorised representatives from the Sponsor, research ethics committee, host institution and the regulatory authorities to permit trial-related monitoring, audits and inspections.

This trial is being conducted in accordance with Good Clinical Practice (GCP), as defined by the International Conference on Harmonisation and in accordance with the ethical principles underlying European Union Directive 2001/20/EC and 2005/28/EC. This trial may be subject to internal or external auditing or inspections procedure to ensure adherence to GCP. Access to all trial-related documents will be given at that time. In accordance with the legislation, the trial master file comprising the essential documents, which enable both the conduct of the trial and the quality of the data produced to be evaluated, will be available to provide the basis for the GCP inspection.

### Dissemination policy

Preliminary study findings may be presented at national and international neuroscience and psychiatry conferences. Final findings will be submitted for publication in relevant peer-reviewed journals. Upon publication, findings may be further publicised in national and international print and electronic media through the Trinity College Dublin and St. Patrick’s University Hospital websites and public relations departments. Authorship eligibility will be based on International Committee of Medical Journal Editors guidelines. There are no plans to use professional manuscript writers. There are no plans for public sharing of participant-level data or statistical code used to generate results. Pseudonymised data can be made available upon reasonable request.

## Discussion

While ketamine as a treatment for depression has attracted a great deal of clinical, academic and general public attention, its role in routine clinical practice has not yet been established. It has often been accompanied by uncritical media attention and sometimes unsubstantiated claims [45]. Additionally, limited data are currently available about the use of serial ketamine infusions, as well as longer-term safety and abuse potential [9].

This protocol describes an ongoing pragmatic trial assessing the real-world effectiveness of serial subanaesthetic ketamine infusions as an add-on treatment for patients hospitalised with depression against an active comparator (midazolam). This trial is also monitoring longer-term therapeutic, safety and tolerability outcomes of repeated ketamine infusions, as well as evaluating cost effectiveness and quality of life throughout the randomised treatment phase and a naturalistic 24-week follow-up. This will help us to clarify a role for serial ketamine in routine clinical practice. It will also provide valuable longer-term safety and effectiveness data, as well as measurement of withdrawal symptoms in order to address concerns about abuse potential.

Since the protocol for this trial was first designed, a more recent meta-analysis of ketamine versus midazolam [12] has reported an effect size of 0.7 in favour of ketamine. Based on this, 44 patients are required per group to achieve 90% power using a two-sided t-test at 5% level. This is in line with the original sample size calculation.

The recruitment for this trial was delayed by the COVID-19 pandemic and the first patient was randomised in September 2021. As of time of writing, 49 eligible participants have been randomised. Recruitment is expected to continue until mid-2024.

## Data Availability

Not applicable. This manuscript does not contain any data.

## Abbreviations

ATRQ: Antidepressant Treatement Response Questionnaire
BPRS: Brief Psychiatric Rating Scale
CADSS: Clinician-administered Dissociative States Scale
CSRI: Client Service Receipt Inventory
CVA: cerebrovascular accident
DMC: Data Monitoring Committee
DSM-5: Diagnostic and Statistical Manual of Mental Disorders, Fifth Edition
ECG: Electrocardiogram
ECT: Electroconvulsive therapy
EQ-5D-5L: EuroQol-5 dimensions-5 level scale for health status
GCP: Good Clinical Practice
IMP: Investigational medicinal product
IV: Intravenous
KARMA-Dep (2): Ketamine as an adjunctive therapy for major depression (2):a randomised controlled trial
MADRS: Montgomery-Åsberg Depression Rating Scale
MINI: Mini-International Neuropsychiatric Interview
MoCA: Montreal Cognitive Assessment
NMDAR: N-methyl-D-aspartate receptor
OAA/S-R: Observer’s Assessment of Alertness/Sedation Scale - Responsiveness Subscale
PRISE: Patient-Rated Inventory of Side Effects
PWC-20: 20-item Physician Withdrawal Checklist
QIDS-SR_16_: Quick Inventory of Depressive Symptoms, Self-Report
STAR*D: Sequenced Treatment Alternatives to Relieve Depression
TSC: Trial Steering Committee
YMRS: Young Mania Rating Scale
